# Physico-Chemical Characterization and Biological Tests of Collagen/Silk Fibroin/Chitosan Scaffolds Cross-Linked by Dialdehyde Starch

**DOI:** 10.3390/polym12020372

**Published:** 2020-02-07

**Authors:** Sylwia Grabska-Zielińska, Alina Sionkowska, Katarzyna Reczyńska, Elżbieta Pamuła

**Affiliations:** 1 Department of Biomaterials and Cosmetic Chemistry, Faculty of Chemistry, Nicolaus Copernicus University in Toruń, 87-100 Toruń, Poland; 2 Department of Physical Chemistry and Polymer Physical Chemistry, Faculty of Chemistry, Nicolaus Copernicus University in Toruń, 87-100 Toruń, Poland; 3 Department of Biomaterials and Composites, Faculty of Materials Science and Ceramics, AGH University of Science and Technology, 30-059 Kraków, Poland; kmr@agh.edu.pl (K.R.); epamula@agh.edu.pl (E.P.)

**Keywords:** biological tests, chitosan, collagen, silk fibroin

## Abstract

In this study, three-dimensional (3D) biopolymeric scaffolds made from collagen, silk fibroin and chitosan were successfully prepared by the freeze drying method. Dialdehyde starch (DAS) was used as a cross-linking agent for the materials. The properties of the materials were studied using density and porosity measurements, scanning electron microscope (SEM) imaging, swelling and moisture content measurements. Additionally, cytocompatibility of the materials in contact with MG-63 osteoblast-like cells was tested by live/dead staining and resazurin reduction assay on days 1, 3 and 7. It was found that new 3D materials made from collagen/silk fibroin/chitosan binary or ternary mixtures are hydrophilic with a high swelling ability (swelling rate in the range of 1680–1900%). Cross-linking of such biopolymeric materials with DAS increased swelling rate up to about 2100%, reduced porosity from 96–97% to 91–93%, and also decreased density and moisture content of the materials. Interestingly, presence of DAS did not influence the microstructure of the scaffolds as compared to non-cross-linked samples as shown by SEM. All the tested samples were found to be cytocompatible and supported adhesion and growth of MG-63 cells as shown by live–dead staining and metabolic activity test.

## 1. Introduction

Bone tissue plays many important functions in the human body, e.g., supporting, transferring load, protecting organs, acting as a reservoir of vital elements and cells [1]. Treatment of bone tissue defects resulting from injuries, infections and diseases is a big challenge for modern medicine [2,3]. In response to the needs of bone tissue engineering, new biomaterials with required features such as biocompatibility, bioresorbability, bioactivity and degradability to non-toxic degradation products have been designed [2,3,4]. Materials based on polymers in the form of porous scaffolds are used to obtain implants for filling small bone tissue cavities [5]. Such scaffolds should be characterized by a combination of chemical and biological properties [6,7,8]. Increasingly, natural polymers are used for this type of biomaterials thanks to their biocompatibility and properties similar to those of extracellular matrix (ECM) [9]. Biomaterials based on natural polymers can be biodegradable due to several chemical moieties in the biopolymer structure [9]. Implants made of them can slowly degrade while reconstituting the tissue. When the biomaterial is biodegradable and the degradation products are non-toxic, the biomaterial does not elicit an intensive immune response and its removal after tissue regeneration is not needed [9].

A number of protein polymers (silk fibroin, collagen, gelatin) and polysaccharides (chitosan, carrageenan, chondroitin sulphate, sodium alginate, hyaluronic acid) are used in biomaterials production [10,11,12,13,14]. Currently, polymer implants are most commonly manufactured with collagen [15,16,17]. Collagen is one of the most abundant proteins in the human body (one third of total proteins) and it is the main protein of the connective tissue. It is responsible for the strength of skin, bones, tendons and cartilage tissue [9]. Nevertheless, collagen materials have several disadvantages, including their poor stability in aqueous environment, because they swell and then dissolve [18,19]. It is therefore necessary to modify collagen-based materials by the addition of different natural or synthetic polymers, or by a cross-linking process [10,20].

Significantly better physio-chemical properties have been demonstrated for silk fibroin [21,22]. Silk fibroin is a biodegradable polymer, biocompatible with human tissues [23]. Materials based on silk fibroin provide good support for cell adhesion and proliferation, without causing cell toxicity [24]. Polysaccharides, such as chitosan, can also be used as a component of different biomaterials [25]. For the production of biomaterials with better properties, mixtures of two or more biopolymers can be used [26,27,28]. In addition, such mixtures can be subjected to cross-linking to improve parameters such as stability in aqueous conditions and regularity of pores [27,29,30]. After the cross-linking process, it is expected that stability in aqueous conditions and resistance to degradation would be improved [27].

Nowadays, many synthetic cross-linking agents including formaldehyde, glutaraldehyde, polyepoxy compound, and hexamethylene-1,6-diaminocarboxysulphonate (HDACS), are used for biomaterial modifications [31,32,33]. Nevertheless, the high or relatively high cytotoxicity of these synthetic cross-linking agents has been reported in the literature, which has confined their applications accordingly [34]. Hence, a new type of cross-linking agent is needed to overcome the cytotoxic effects of the synthetic cross-linkers and, simultaneously, endow biomaterials with superior physicochemical properties.

Dialdehyde starch (DAS) has been used for cross-linking of the scaffolds for tissue engineering [29]. DAS is a high molecular weight cross-linker. It is more environmentally friendly and less toxic than other aldehyde compounds with low molecular weights [29]. The aldehyde groups from DAS might react with the free amino groups within collagen, chitosan and silk fibroin molecules, which was demonstrated for collagen with alginate dialdehyde [35]. The formation of cross-linking bonds between aldehyde groups from DAS and amine groups of proteins was demonstrated in the article by J. Skopinska-Wiśniewska et al. [29].

The aim of this work was to design new materials of silk fibroin, collagen and chitosan and to obtain polymeric three-dimensional (3D) matrices based on mixtures of these polymers. Two-component collagen and chitosan mixtures with organic and inorganic additives have been described previously [36]. Binary blends based on collagen and silk fibroin have been prepared and their physico-chemical properties and microstructure were studied [21,37]. Mixtures of collagen/silk fibroin with the addition of inorganic particles [38] and cross-linking agents [39] have also been described in the literature. Films [21,40,41] and 3D scaffolds [42] based on chitosan and silk fibroin were studied. The combination of silk fibroin, collagen and chitosan is a novel approach to the creation of biomaterial that can be potentially used in bone tissue engineering.

In this study, three-component materials based on collagen, silk fibroin and chitosan were obtained and their porosity, density, morphology, swelling ability and moisture content were studied. The influence of DAS as a cross-linking agent on properties of the obtained scaffolds was investigated. Biological tests were carried out to study the effect of the obtained materials on osteoblast-like cells.

## 2. Materials and Methods

### 2.1. Materials

Chitosan (CTS) was supplied by Sigma-Aldrich (Poznań, Poland). The deacetylation degree (DD, %) of chitosan was 78%, and the viscosity average molecular weight was 0.59 × 10^6^ g/mol.

Collagen (Coll) was extracted in our laboratory, from rat tail tendons [21,25]. The tendons were excised and washed in distilled water, and dissolved in 0.1 M acetic acid for three days at 4 °C. The undissolved parts were removed by centrifugation for 10 min at 10,000 rpm. The obtained solution was frozen at −18 °C and lyophilized at −55 °C and 5 Pa for 48 h (ALPHA 1–2 LD plus, CHRIST, Germany). Chitosan and collagen were prepared as 1% solution in 0.1 M acetic acid.

Silk fibroin (SF) was also prepared in our laboratory. It was extracted from *Bombyx mori* cocoons (Jedwab Polski Sp. z o.o., Milanówek, Poland). Cocoons were boiled twice for 1 h in aqueous solution of 0.5% Na_2_CO_3_, according to the procedure found in the literature [21,40]. After that, the cocoons were boiled in 5% alkaline soap solution for 30 min and then for 20 min in distilled water to extract the sericin proteins. This procedure was repeated three times. After that, the prepared silk fibroin was dried at room temperature. Then, the silk fibroin was dissolved in 9.3 M lithium bromide. Dissolution was carried out at 80 °C for 4 h. It was prepared as a 5% concentrated solution. Then the solutions were filtered and dialyzed against distilled water for 3 days to yield fibroin aqueous solutions.

Dialdehyde starch (DAS) (potato origin, *M*v = 188 g/mol) was purchased from Chemos GmbH&Co. KG (Altdorf, Germany). The aldehyde group content in DAS was determined by the alkali consumption method and equalled 67% [43]. Dialdehyde starch solution at 3% concentration in distilled water was prepared.

Chitosan and silk fibroin were mixed at the 50/50 wt ratio with the use of a magnetic stirrer for 1 h. Chitosan was added in 25 *w*/*w* % addition during mixing. Then, DAS was added in 10 *w*/*w* % ratio and mixing was continued for the next 2 h. The mixtures were poured into 24-well polystyrene culture plates, frozen, and lyophilized (ALPHA 1–2 LDplus, CHRIST, −55 °C, 5 Pa, 48 h).

### 2.2. Porosity and Density

Porosity and density were measured using liquid displacement method with isopropanol as the liquid, because it did not dissolve the samples [44]. Porous sample with a known weight (*W*) was immersed for 3 min in a cylinder with a known volume of isopropanol (*V*_1_). The volume of isopropanol with the sample was measured (*V*_2_) and the volume of liquid after the removal of scaffold (*V*_3_) was measured too. The porosity of the scaffold (ε) was calculated using Equation (1), and the density (*d*) was calculated using Equation (2):

(1) $$\varepsilon =\frac{V_{1}-V_{3}}{V_{2}-V_{3}}\times 100\%\;\left[\%\right]$$

(2) $$d=\frac{W}{V_{2}-V_{3}}\;\left[\frac{mg}{cm^{3}}\right]$$

$V_{1}$—initial volume of isopropanol [cm^3^],
$V_{2}$—total volume of isopropanol and isopropanol impregnated scaffold [cm^3^],
$V_{3}$—isopropanol volume after sample removing [cm^3^],
W—sample weight [mg].

Samples of each kind were measured in triplicate.

### 2.3. Microstructure of the Scaffolds

The microstructure of the samples was studied using Scanning Electron Microscope (SEM) (LEO Electron Microscopy Ltd., England, UK). The scaffolds were frozen in liquid nitrogen for 3 min. Freezing of a sample allows its gentle cutting with a razor scalpel to observe its interior microstructure. The samples were sputter-coated with a layer of gold prior to observation. The size of the pores was measured on SEM images using the Sigma Scan Pro 5 software [45,46,47]. To determine the size of pores at least 10 pores were measured.

### 2.4. Swelling Behaviour and Moisture Content

The swelling ratio was measured by immersing the composites’ fragments in phosphate buffered saline (PBS) solution, pH = 7.4. After 1 h of immersion, the materials were gently dried by putting them between two sheets of paper, and then weighed [21]. The swelling ratios were calculated using the Equation (3):

(3) $$swelling=\frac{\left(m_{t}-m_{0}\right)}{m_{0}}\times 100\%\;\left[\%\right]$$

$m_{t}$—weight of the material after immersion in PBS [mg],
$m_{0}$—weight of the material before immersion [mg].

Scaffold moisture contents were determined by drying samples in an oven at 105 °C until they reached a constant weight. The results were expressed as grams of water in 100 g of dry sample weight. Samples of each kind were measured in triplicate.

### 2.5. Biological Tests

Cytocompatibility of the materials was tested using MG-63 osteoblast-like cells (European Collection of Cell Cultures, Salisbury, UK). The cells were cultured in Eagle’s minimal essential medium (EMEM, PAN BIOTECH, Aidenbach, Germany) supplemented with 10% foetal bovine serum, 1% penicillin-streptomycin and 0.1% sodium pyruvate (PAA, Pasching, Austria). The cells were cultured at 37 °C, in a humidified atmosphere containing 5% CO_2_. Prior to cell culture, the scaffolds (2–4 mm in height, 15 mm in diameter) were sterilized by soaking in 70% ethanol aqueous solution and washed five times with sterile PBS (pH = 7.4, PAA, Paschin, Austria). The scaffolds were then transferred to 24-well plates and seeded with 1 × 10^4^ cells per sample (suspended in 1 mL of EMEM). The cells seeded directly on tissue culture polystyrene (TCPS, Nunclon) served as control. The experiment was run in triplicate.

After 1, 3 and 7 days, the viability of the cells was determined using resazurin reduction assay. Cell culture medium was carefully removed from the wells and replaced with 1 mL of fresh cell culture medium containing 5% of AlamarBlure reagent (In Vitro Toxicology Assay Kit, Resazurin based, Sigma Aldrich, St. Louis, MO, USA). After 3 h of incubation, 100 µL of the medium was transferred into a black 96-well plate for fluorescence measurement (λ_ex_ = 544 nm, λ_em_ = 590 nm, FluoStar Omega, BMG Labtech, Ortenberg, Germany). The percentage of resazurin reduction was calculated according to the following Formula (4):

(4) $$Reduction\;of\;resazurin=\frac{S^{x}-S^{control}\;}{S^{100\%reduced}-S^{control}}\times 100\%\;\left[\%\right]$$

$S^{x}$—fluorescence of the samples,
$S^{control}$—fluorescence of EMEM with 5% AlamarBlue reagent but without cells (0% reduction of resazurin),
$S^{100\%reduced}$—fluorescence of EMEM with 5% AlamarBlue reagent autoclaved for 15 min at 121 °C (100% reduction of resazurin).

Cell attachment, spreading and viability were also evaluated using fluorescence microscopy after live/dead staining. The samples were carefully washed with PBS and stained using 0.1% calcein AM (Sigma-Aldrich) and 0.1% propidium iodide (Sigma-Aldrich) dissolved in PBS for 20 min at 37 °C. Fluorescence microscopy images were taken using Axiovert 40 (Zeiss, Oberkochen, Germany) microscope with HXP 120 C Metal Halide Illuminator (Zeiss, Oberkochen, Germany).

## 3. Results and Discussion

### 3.1. Porosity and Density

Porosity and density are very important parameters, which have to be defined for porous scaffolds for tissue engineering. The porosity and density were measured for single polymers (collagen, silk fibroin) and their mixtures with the addition of chitosan and a cross-linking agent (DAS). The results are presented in Table 1. The lowest porosity was found for Coll (88 ± 0.5%) and it did not change significantly upon cross-linking with DAS (89 ± 4.0%). However, for all other samples porosity decreased after addition of DAS. The porosity of all cross-lined hydrogels was in range of 86–91%. Scaffolds for bone tissue regeneration should mimic bone morphology, function and structure in order to optimize integration with surrounding tissue. As it is well known, the appropriate porosity of a material for bone tissue engineering applications should be about 90%, as this allows sufficient nutrient and gas exchange as well as provides enough space for cell attachment and proliferation [48,49,50,51].

Decrease in material porosity upon cross-linking with DAS was accompanied by an increase in sample density in all samples, except Coll, in which density, like porosity, was not affected by the presence of DAS. The most pronounced differences in both porosity and density were observed for SF, which may indicate strong affinity of DAS to SF. The concentration of SF in SF/Coll and SF/Coll/CTS mixtures was lower, thus the influence of DAS was less distinct. Increasing density of material would favour cell growth and proliferation, as more materials can be accessed by the cells [50,51]. Increased matrix density enhances proliferation due to an increase in matrix stiffness [50]. Increase in matrix stiffness accompanies increased density [51].

### 3.2. Microstructure of the Scaffolds

**Figure 1:**
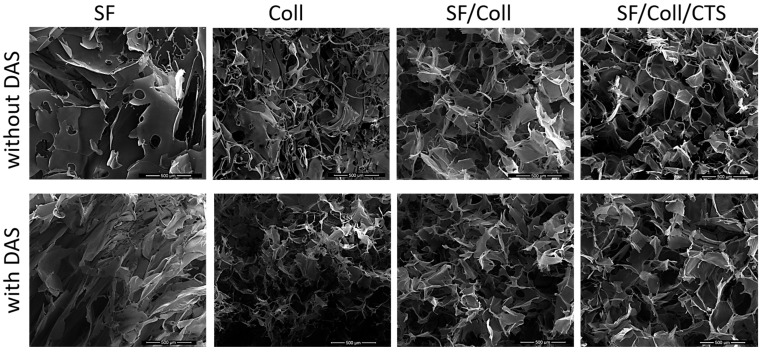
SEM images of every scaffolds with scale bar 500 µm.

**Figure 2:**
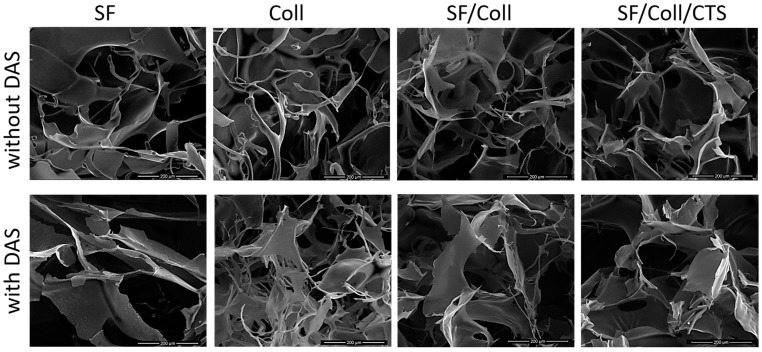
SEM images of every scaffolds with scale bar 200 µm.

The morphology of the scaffolds was evaluated with scanning electron microscopy (Figure 1 and Figure 2). Each scaffold had a porous structure with interconnected pores. Pores with a diameter of ca. 200 μm or smaller were present. The size of the pores was determined based on SEM images [45,46]. The pores had similar sizes to pores in scaffolds made of chitosan, collagen and hyaluronic acid [43]. Such a material microstructure is necessary for the tissue engineering purposes [44]. According to Hulbert et al., the minimum pore size required to bone tissue regeneration is generally considered to be ~100 μm [52]. However, larger pores of 100–200 μm showed substantial bone ingrowth as well. Smaller pores (75–100 μm) resulted in ingrowth of unmineralized osteoid tissue, and the smallest pores (10–75 μm) were penetrated only by fibrous tissue [52]. These results were correlated with normal Haversian systems that reach an approximate diameter of 100–200 μm [52]. The larger pore size allows a greater number of blood vessels to grow [45]. The minimum recommended pore size for scaffolds is 100 μm according to the early work of Hulbert et al. [52], but subsequent studies have shown better osteogenesis for implants with pores that are >300 μm [53,54,55]. For materials made of silk fibroin, bigger pores can be observed than in materials made of collagen. It has been shown by other research groups that, for silk fibroin and collagen mixture scaffold, the changes in structure and morphology in comparison with scaffolds made of one biopolymer can be observed [56]. The results of our previous paper [39] and this research on SF/Coll and SF/Coll/DAS are consistent with the previous paper regarding the SF/Coll blend. The microstructure of ternary mixture material was more uniform, and more regular pores were observed. After the addition of a cross-linking agent, the largest changes were observed for SF/Coll and SF/Coll/DAS. The microstructure of modified scaffold is more organized comparing with non-crosslinked sponges (especially for collagen scaffold) as in our previous research [39]. The addition of dialdehyde starch improves the organization of sponges as has been shown by Kaczmarek et al. [57] for when the sponges were made of gelatin and chitosan with 5% addition of DAS. However, significant changes were not observed for scaffolds based on binary and ternary mixtures, likewise, in our earlier research, when chitosan and collagen were mixed with sodium alginate [58] and when collagen, chitosan and hyaluronic acid were mixed together to form ternary blend [59,60]. 

### 3.3. Swelling Behaviour and Moisture Content

The materials made of collagen, silk fibroin and chitosan were easily wettable by polar solvents such as PBS. They exhibited a high swelling ability. It is because collagen, chitosan and silk fibroin contain a large number of functional groups capable of binding water [21,61,62]. The fast-swelling behaviour is a characteristic property of hydrophilic and porous materials [44]. PBS solution has a pH = 7.4, which corresponds to the pH of blood. The use of such a solution allows the examination of the behaviour of the material after its application inside the body [44]. The percentage of scaffold swelling after the immersion in PBS for 1 h, together with the results of moisture content measurements, are presented in Table 2.

No result for collagen can be found in Table 2, because after a few minutes in PBS, the scaffolds dissolved. During 1 h of incubation, all the scaffolds, excluding Coll/DAS, absorbed around 1500–2100% of PBS. For materials cross-linked with DAS, an increase in swelling degree by a factor of 200–300% was observed [61,62]. The swelling ability for scaffolds made of the binary and ternary blend was almost twice those in the scaffolds made of collagen with DAS (2091 ± 136% for SF/Coll/DAS, 2102 ± 9% for SF/Coll/CTS/DAS compared to 1084 ± 48% for Coll/DAS). Interestingly, the swelling of hydrogel mixtures was higher than the swelling of their single components. This indicates that there are some specific interactions between hydrogels and cross-linking molecules, which need to be fully evaluated in further research. Our observations are in line with other research on binary and ternary blends (chitosan, collagen and hyaluronic acid) cross-linked by DAS [59,62]. It was also observed that the addition of collagen to silk fibroin increased the swelling ability of the material; the same as with the addition of chitosan to silk fibroin [42]. The highest moisture content was found for SF/Coll/CTS scaffolds (22.43 ± 1.32 g/100 g), while the lowest was for the SF sample (only 7.09 ± 1.03 g/100 g). Generally, the moisture content decreased for materials with the addition of DAS. However, that did not apply to samples made of pure collagen; moisture content for Coll and Coll/DAS was at similar level (14.17 ± 1.36 and 13.10 ± 0.79 g/100 g, respectively).

### 3.4. Biological Tests with Osteoblast-Like MG-63 Cells

Developed scaffolds are intended to be implanted into small bone tissue defects. For that, the scaffolds have to be non-toxic and facilitate bone cell adhesion and proliferation. To assess biological properties of the developed materials, they were seeded with osteoblast-like MG-63 cells [63].

**Figure 3:**
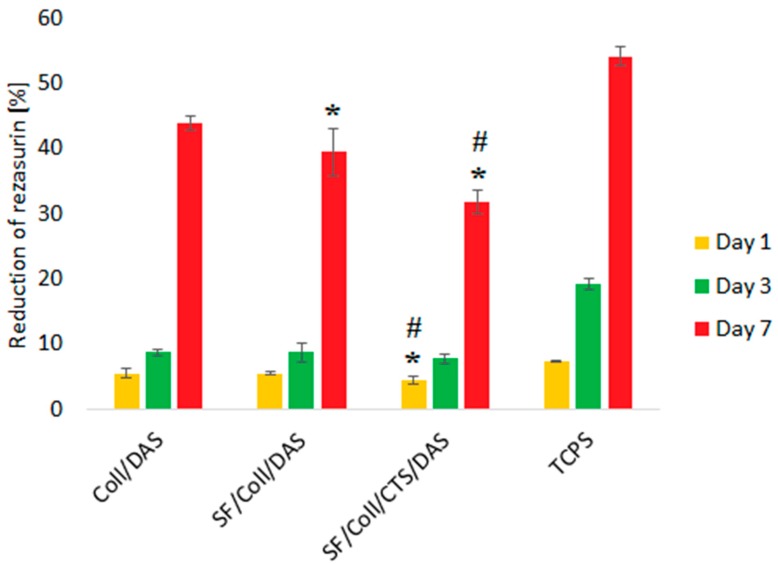
Metabolic activity reflected by reduction of resazurin on days 1, 3, 7 of MG-63 cells cultured on Coll/DAS, SF/Coll/DAS and SF/Coll/CTS/DAS scaffolds and on control TCPS. Mean ± standard error of the mean (* *p* < ± 0.05 vs. Coll/DAS; # *p* < ± 0.05 vs. SF/Coll/DAS according to one-way ANOVA with Holm–Sidak post hoc test).

The results of the biological test were shown only for three types of the samples, i.e., those cross-linked with DAS, because the samples without cross-linking agents were dissolved after 1 day of cell culture. Also, the SF scaffold was dissolved in cell culture medium. Therefore, the positive effect of DAS on the stability of the samples made exclusively of collagen, as well as ones made of binary and ternary blend materials in cell culture medium can be clearly observed. Metabolic activity of MG-63 cells was measured after 1, 3 and 7 days (Figure 3). Regardless of the sample type, metabolic activity of the cells, which indirectly represents their proliferation, increased as a function of culture time. The fastest proliferation was observed for Coll/DAS, but by the end of the experiment (7 days), the metabolic activity of the cells cultured on Coll/DAS was similar to those cultured on SF/Coll/DAS. Slower proliferation of the cells was observed in the case of SF/Coll/CTS/DAS. The cells cultured on TCPS showed the highest reduction of resazurin, as TCPS is regarded as an ideal substrate for cell adhesion and proliferation. It must be kept in mind that in the case of TCPS, all of the cells initially seeded remained in the well and their activity was measured throughout the experiment. However, once the cells were seeded on the scaffolds, some of the cells did not attach to the scaffolds, but settled at the bottom of the well as the scaffolds were slightly smaller than the well itself. Prior to testing cell metabolic activity, the scaffolds were transferred into a new plate, so that only the cells growing on the scaffolds were evaluated.

**Figure 4:**
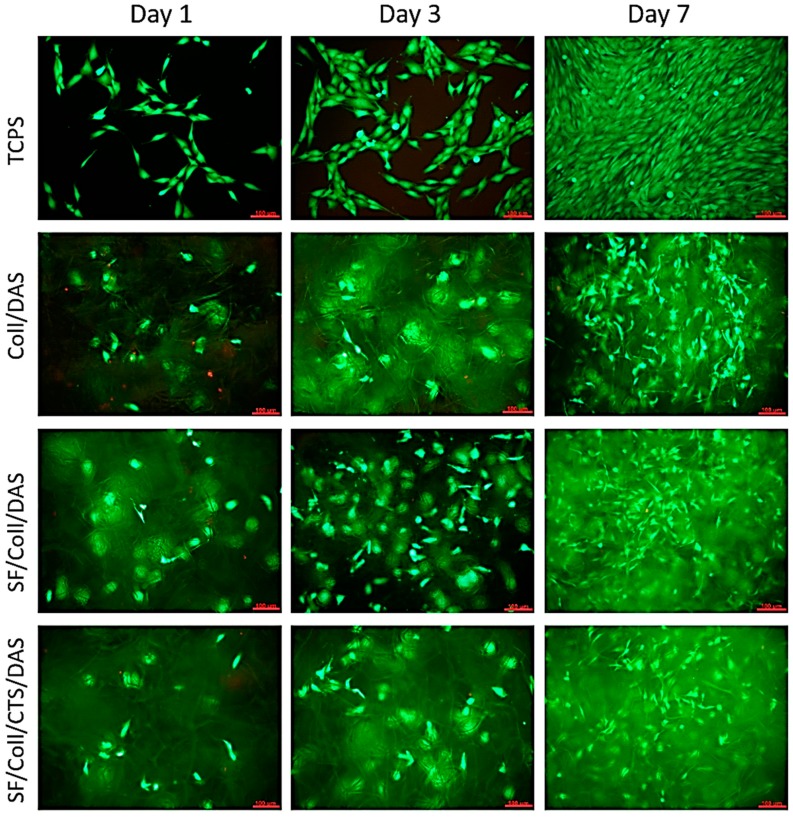
Images of MG-63 cells grown for 1, 3 or 7 days on Coll/DAS, SF/Coll/DAS and SF/Coll/CTS/DAS scaffolds and on control TCPS. Live/dead staining. Scale bar = 100 μm.

The results of metabolic activity were further confirmed by a live/dead assay. Figure 4 shows the morphology of the cells stained in green (live cells) and red (dead cells). The results show that the vast majority of the cells were alive and, in all cases, there were less than 2% dead cells.

At the beginning of the culture, the number of cells visible on the samples was low, but it gradually increased as the culture time increased. At all time points, live cells were visible on the samples. The distribution of the cells was uniform and migration of some cells into deeper parts of the scaffolds was observed. After 7 days of culture, their morphology was similar to those of the cells cultured on control TCPS. The cells were characterized by an elongated, spindle shape. In the case of the Coll /DAS sample, material degradation occurred very quickly. After 3 days of culture, small fragments of the material detached from the sample very easily, and the sample itself, after being removed from the culture well, was collapsed. Slower, but still visible material degradation also occurred in the other two samples. For this reason, it is difficult to assess whether the observed differences in the metabolic activity of the cells cultured on the tested materials resulted directly from the cytotoxicity of a given material, or whether it was the result of a decrease in the volume or surface of the sample available to the cells.

All in all, the analysed samples were cytocompatible with MG-63 cells. Cells grown on these materials proliferated normally and showed morphology similar to control cells. The number of cells growing on the material increased with time and after 7 days of culture, the cells evenly covered almost all the available surface of the material. Our results are consistent with previous studies pointing out that the addition of DAS to chitosan/gelatin scaffolds supported SaOs-2 cell attachment and proliferation without causing any cytotoxicity effects [57]. In other studies, it was shown that the addition of even up to 5% of DAS did not reduce cell viability [64].

The use of other commonly applied chemical cross-linking agents, such as formaldehyde, glutaraldehyde, or glyceraldehyde is limited due to their possible toxicity and leakage of unreacted cross-linker molecules in vivo. Thus, novel and safer cross-linking compounds e.g., genipin are being extensively investigated [65]. Our research showed that DAS is a promising alternative for genipin, as it is non-toxic and much less expensive.

## 4. Conclusions

Three-dimensional scaffolds based on silk fibroin, collagen and chitosan, cross-linked by dialdehyde starch were obtained by a freeze drying method. They were characterized by high swelling degree, high porosity (about 90%) and interconnected pores less than 200 µm in size. The addition of a cross-linking agent (DAS) modified physico-chemical properties of the materials. These materials had better swelling ability, which is important for tissue engineering applications. All the studied materials were cytocompatible with MG-63 cells. It can be assumed that the scaffolds based on silk fibroin, collagen and chitosan, cross-linked by DAS can be proposed as potential biocompatible matrices for tissue engineering purposes. It is expected that they will promote bone healing and regeneration.

## Figures and Tables

**Table 1:** Porosity and density of the scaffolds.

Sample	Porosity [%]		Density [mg/cm^3^]	
	without DAS	with DAS	without DAS	with DAS
SF	95 ± 1.7	86 ± 4.0	33.0 ± 0.4	34.6 ± 2.4
Coll	88 ± 0.5	89 ± 4.0	16.9 ± 2.2	16.0 ± 1.4
SF/Coll	97 ± 1.0	91 ± 0.9	14.8 ± 2.1	17.7 ± 0.4
SF/Coll/CTS	96 ± 1.9	93 ± 1.9	17.0 ± 1.5	19.0 ± 0.7

**Table 2:** Scaffolds swelling ratio and moisture content after immersion in PBS for 1 h; ^Nd^—not determined.

Sample	Swelling Ratio [%]		Moisture Content in 100 g of Dry Sample [g]	
	without DAS	with DAS	without DAS	with DAS
SF	1511 ± 147	1792 ± 93	7.09 ± 1.03	12.09 ± 0.50
Coll	^Nd^	1084 ± 48	14.17 ± 1.36	13.10 ± 0.79
SF/Coll	1891 ± 236	2091 ± 136	19.27 ± 0.92	17.33 ± 0.30
SF/Coll/CTS	1678 ± 19	2102 ± 9	22.43 ± 1.32	19.60 ± 1.35
